# Comparative efficacy and safety of glyburide, metformin, and insulin in treatment of gestational diabetes mellitus: A protocol for systematic review and meta-analysis

**DOI:** 10.1097/MD.0000000000029090

**Published:** 2022-03-18

**Authors:** Jing Lin, Rong-zu Tu, Xun-yu Hong

**Affiliations:** ^a^ *Department of Health Management Center, Ningbo Women and Children’s Hospital, Zhejiang, China,* ^b^ *Department of Education, Ningbo Women and Children’s Hospital, Zhejiang, China,* ^c^ *Department of Internal Medicine, Ningbo Women and Children’s Hospital, Zhejiang, China.*

**Keywords:** gestational diabetes mellitus, glyburide, insulin, meta-analysis, metformin

## Abstract

**Background:**

The increased prevalence of gestational diabetes mellitus (GDM) has caused a huge societal economic and healthy burden at both the population and individual levels. We aimed to assess the comparative efficiency and safety of the use of glyburide, metformin, and insulin in GDM from a protocol for systematic review and meta-analysis.

**Methods:**

Two individual researchers conducted the platform searches on the PubMed, Cochrane Library, and Embase databases from inception to February 2022. Literature retrieving was carried out through a combined searching of subject terms (“MeSH” on PubMed and “Emtree” on “Embase”) and free terms on the platforms of PubMed and Embase, and through keywords searching on platform of Cochrane Library. Systematic review and meta-analysis of the data will be performed in STATA13.0 software according to the Preferred Reporting Items of Systematic Reviews and Meta-Analysis (PRISMA) guidelines. Two authors independently performed the literature searching, data extraction, and quality evaluation. Risk of bias was assessed using the Cochrane Risk of Bias Tool for randomized controlled trials.

**Results:**

The results will be submitted to a peer-reviewed journal.

**Conclusion:**

This meta-analysis will provide a comprehensive analysis and synthesis that can be used as an evidence map to inform practitioners and policy makers about the effectiveness of glyburide, metformin, and insulin for patients with GDM.

## 1. Introduction

Gestational diabetes mellitus (GDM) is one of the common metabolic complication during pregnancy and is associated with an increased risk of adverse pregnancy outcomes for both mothers and their offspring not only in the short-term but also in the long-term.^[1-3]^ Women with GDM are at an increased risk of perinatal morbidity. These women are also at particular high risk of diabetes and cardiovascular disease in their later life. According to a meta-analysis of 20 studies and 1,332,373 individuals (67,956 women with GDM and 1,264,417 controls), women with a history of GDM have a nearly 10-fold higher risk of developing type 2 diabetes mellitus than those with normoglycemia during pregnancy.^[4]^ The increased prevalence of GDM has caused a huge societal economic and healthy burden at both the population and individual levels.^[5,6]^

Insulin historically has been considered the standard therapy for GDM management in cases refractory to nutrition therapy and exercise. However, it requires multiple daily injections and subsequently the need to train the patients in the technical aspect of treatment, resulting to more weight gain and higher medical cost. In addition, hypoglycemia occurs in approximately 70% of women who use insulin some time during their pregnancy.

Metformin, as the first-line medication for type 2 diabetes mellitus, can promote glucose level control and lose weight and improve peripheral insulin resistance.^[7]^ Metformin is also known to increase the secretion of glucagon-like peptide 1 from intestinal cells. It is increasingly recognized as an alternative to insulin therapy for GDM.^[8]^ However, metformin has been found to have a maternal to fetal transfer and the long-term influence is uncertain. Glyburide can stimulate the release of insulin from the pancreas and it is a second-generation sulfonylureas that can be considered safe and effective for the treatment of GDM.^[9]^ At present, there are some concerns regarding a higher risk of macrosomia, large-for-gestational age infants, and neonatal hypoglycemia compared to insulin.^[10]^ Based on the controversy above, we performed a protocol for systematic review and metaanalysis to evaluate the efficacy and safety of glyburide, metformin, and insulin in treatment of GDM.

## 2. Methods

### 2.1. Protocol register

This protocol of systematic review and meta-analysis has been drafted under the guidance of the preferred reporting items for systematic reviews and meta-analyses protocols.^[11]^ It has been registered on open science framework (registration number: 10.17605/OSF.IO/MWCDR). Ethical approval is not required for this study since it relies on secondary data.

### 2.2. Search strategy

Two individual researchers conducted the platform searches on the PubMed, Cochrane Library, and Embase databases from inception to February 2022. Literature retrieving was carried out through a combined searching of subject terms (“MeSH” on PubMed and “Emtree” on “Embase”) and free terms on the platforms of PubMed and Embase, and through keywords searching on platform of Cochrane Library. References within included articles were reviewed to include articles that were not included within our literature search. The key terms used for the search were “glyburide”, “metformin”, “insulin”, and “gestational diabetes mellitus”. The retrieval process is presented in Figure 1.

**Figure:**
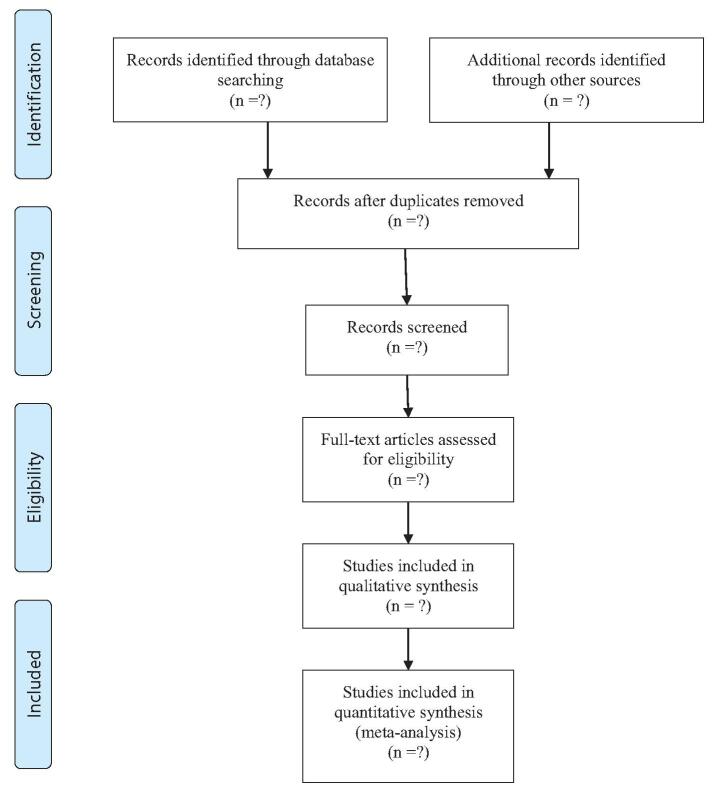
**Figure 1.** Flow diagram of study selection.

### 2.3. Inclusion and exclusion criteria

Studies were included if they met the following criteria: subjects were women with GDM requiring drug treatment; the study was a randomized controlled trial that compares efficacy and safety parameters of metformin, glyburide, or insulin; the study provided information on 1 or more maternal or fetal outcome. The exclusion criteria were as follows: reviews, letters, conferences abstract, case reports or series, comments, and animal experiment. Studies involving pregnant women with pre-existing diabetes and studies with duplicated data were also excluded.

### 2.4. Data extraction

The 2 authors extracted valid data from the included original literature and reviewed the data extracted by each other for accuracy. The data extracted from the studies included title, published year, authors, country, study design, sample size, sex distribution, the type of interventions, and duration of follow-up. Differences and disagreements were settled by discussion, the third author was consulted if they cannot reach an agreement

### 2.5. Risk of bias assessment

The methodological qualities of the included studies were assessed independently by the 2 reviewers according to the Cochrane Collaboration’s bias evaluation criteria.^[12]^ Its specific contents include the following 6 aspects: the generation of random methods, the allocation of hidden methods, blind methods, lost follow-up and withdrawal, selective reporting results, and other biases. Grading of Recommendations Assessment, Development, and Evaluation (GRADE) was used to assess the quality of evidence, which was classified as high, moderate, low, or very low. Assessments included the risk of bias, inconsistency, indirectness, impreciseness, and other considerations.

### 2.6. Statistical analysis

Two researchers respectively entered the data into the STATA13.0 software. Mean differences with a 95% confidence interval were calculated to assess the effect size for continuous outcome data. Risk ratio with a 95% confidence interval were used as effect size for dichotomous data. Inverse variance method and Mantel-Haenszel analysis method were used for continuous variables and dichotomous variables, respectively. The heterogeneity among the trials was assessed for significance with Q and quantified with I^2^. Statistically significant was set at the *P* value < .10. If the studies were homogeneous or the statistical heterogeneity was low, we used the fixed effect-model. While, random-effects model was applied when the statistical heterogeneity was moderate or high.

## 3. Discussion

GDM has become an epidemic and caused a tremendous healthy and economic burden. The prevalence of GDM has continued to increase during the past few decades and is likely to see a further rise in the future. The rising prevalence of GDM is partly due to the concurrent increase in well-established risk factors, including advanced maternal age, pre-pregnancy overweight or obesity, excessive gestational weight gain, history of GDM, changes in dietary patterns and lifestyles, etc.^[13]^ Poorly managed GDM can lead to maternal and neonatal adverse outcomes in both the short and long term. The maternal complications include preeclampsia, cesarean section, and polyhydramnios in the short term, and the progression of diabetes mellitus after pregnancy in the long term.^[14,15]^ The fetal and neonatal complications include congenital malformation, neonatal death, stillbirth, macrosomia, obstetric trauma, shoulder dystocia, and neonatal hypoglycemia.^[16,17]^ This meta-analysis will provide a comprehensive analysis and synthesis that can be used as an evidence map to inform practitioners and policy makers about the effectiveness of pharmacological interventions for patients with GDM. Future studies should consider pharmacological diversity by including subgroup meta-analyses or performing meta-regression, as opposed to pooled meta-analyses.

## Author contributions

**Conceptualization:** Rong-zu Tu.

**Data curation:** Rong-zu Tu, Xun-yu Hong.

**Funding acquisition:** Jing Lin.

**Investigation:** Xun-yu Hong, Rong-zu Tu.

**Methodology:** Xun-yu Hong.

**Writing** - **original draft:** Jing Lin.

**Writing** - **review & editing:** Jing Lin.
